# Infection with brainworm (*Elaphostrongylus rangiferi*) in reindeer (*Rangifer tarandus* ssp.) in Fennoscandia

**DOI:** 10.1186/s13028-020-00524-4

**Published:** 2020-05-27

**Authors:** Rebecca K. Davidson, Torill Mørk, Karin E. Holmgren, Antti Oksanen

**Affiliations:** 1 Research: Food Safety and Animal Health, Norwegian Veterinary Institute, Stakkevollvegen 23b, 9010 Tromsø, Norway; 2 Finnish Food Authority (FINPAR), Oulu, Finland

**Keywords:** Climate change, Diagnosis, Epizootic, Goat, Lifecycle, Neurological disease, Pathogenesis, Protostrongylid, *Rangifer tarandus tarandus*, Treatment, Sheep

## Abstract

Sami reindeer herders have considerable traditional knowledge about a neurological reindeer disease resembling elaphostrongylosis, but the causative agent was not identified prior to the description of the brainworm *Elaphostrongylus rangiferi* in Russia in 1958. Elaphostrongylosis was quickly recognised as a serious cause of reindeer morbidity and mortality. The ecology, epidemiology and pathophysiology of the disease were studied in Sweden and Norway during the 1960s and in particular the 1970s to 1990s. In Finland, elaphostrongylosis was not recognised as an important disease for Finnish reindeer husbandry, even though the presence of brainworm infection has been documented. Brainworm has an indirect lifecycle with snail and slug intermediate hosts. The free-living L1 larvae have extremely good freeze tolerance and can survive > 360 days at − 80 °C in water (solid ice). Even though reindeer brainworm is clearly well adapted to the Arctic chill, the lifecycle stages outside the reindeer final host are sped up at warmer environmental temperatures. Arctic summer temperatures are close to the developmental threshold of the parasite in the intermediate gastropod hosts (8–10 °C), and the parasite has typically had a 2-year life cycle. Disease outbreaks generally occur during the winter following the infection of reindeer with infected snails and slugs during the summer and autumn. Warmer summers result in faster development of brainworm larvae in the intermediate hosts. Clinical symptoms have been seen reported as early as August, such as in the outbreak in Trøndelag, Norway in 2018. The reindeer brainworm is also a cause of conflict between reindeer herders and small ruminant farmers, because it can cause severe disease in goats and sheep, which share pasture with reindeer. Many knowledge gaps remain if we wish to successfully predict and mitigate for large-scale outbreaks in a future with a predicted warmer, wetter and wilder climate.

## Background

*Elaphostrongylus rangiferi* (brainworm or meningeal worm) is considered a ubiquitous parasite in reindeer (*Rangifer tarandus* ssp.) in Fennoscandia, Russia and in imported reindeer in Newfoundland, Canada [1, 2]. The parasite was first described in scientific literature in 1958 from reindeer in northern Russia (USSR) [3]. Large outbreaks were subsequently reported in Fennoscandia in the 1960s and early 1970s prompting considerable research efforts into identifying the lifecycle, epidemiology and pathogenesis. Traditional herder knowledge combined with scientific investigations confirmed the seasonality and the highly temperature dependent development stage in snail intermediate hosts of this parasite. But since the heyday of this research, the parasite has almost faded into obscurity and much of knowledge acquired half a century ago hidden in difficult to obtain scientific reports. In this literature review we will explore this knowledge and, combined with more recent findings, discuss why this parasite is of increasing importance and which knowledge gaps still need urgent attention.

## Search strategy

This literature review is based upon searches in PubMed (https://www.ncbi.nlm.nih.gov/pubmed/) and Google Scholar (https://scholar.google.com/) using terms *Elaphostrongylus*, elaphostrongylosis, brainworm, meningeal worm, cerebral nematodiasis and reindeer; as well as searches in grey literature. The authors of this review have, together, more than 60 years of experience in the field of reindeer health and diseases, parasitic diagnostics and control.

## Review

### About reindeer in Fennoscandia

Fennoscandian reindeer are not a homogenous population: from the wild tundra reindeer of central Norway and the semi-domesticated herds (*Rangifer tarandus tarandus*) of the Sami people to the forest reindeer of Finland (*R. tarandus fennicus*) [4, 5]. Reindeer herding in this region is based on traditional nomadic pastoralism of the Sami people. Today there are large variations in production systems within and between countries but still with a focus on the movement of herds between different seasonal grazing areas. Movement of herds across regional, and sometimes national, borders was central to this peripatetic lifestyle but is now governed by national legislation in each country [5]. The Norwegian-Finnish border was, for example, first closed in 1826 and prevents reindeer from grazing in the neighbouring country [6]. Geographical differences in herding practices exist partly as a result of the availability of nature-based resources and, partly, in response to the need to adapt to external pressures and challenges. The fencing of animals and supplying of supplemental feeding in response to pasture loss, predation pressure, and climate events leading to locked grazing, are increasingly practiced, especially in Finland [7]. In Norway, reindeer herding covers 70% of the land usage, but with large geographical variation in reindeer numbers; 70% of the herded reindeer are found in the northernmost county of Finnmark [8].

### Traditional Sami knowledge regarding brainworm

Whilst the parasite was not officially recognised in the scientific community until the late 1950’s, its presence is not new. Sami reindeer herders were well aware of a range of neurological disease signs such as “liwdsa-vikke/livzzavihki” (shaking disease; posterior paresis) and “gattsjadam-vikke” (falling disease) [9–11]. In fact, “livzzavihki” was first described in a 1910 book about the life of the Lapps written by Johan Turi (Muitalus sámiid birra). He describes the disease as causing emaciation and posterior paresis and, sometimes, the death of several hundred reindeers during a short period (Turi J. An account of the Sámi 2010). “Oiave-vuorre” (brain madness) and “kinalkaesahtahka” (stiff neck) in addition to “liwdsa-vikke” are also listed as names, by Qvigstad in 1941, for a range of neurological symptoms seen by reindeer herders [11, 12]. Trauma, other infections of the central nervous system (CNS), metabolic disorders, and heavy throat bot (*Cephenemyia trompe*) infections, are differential diagnoses for such clinical signs. Herders reported that larger animals were most affected and that the disease occurred during winter in all age groups but with calves and yearlings particularly predisposed [9, 10, 12]. They also reported that disease outbreaks were more common after particularly warm summers [10].

### Lifecycle of *E. rangiferi*

**Fig. 1:**
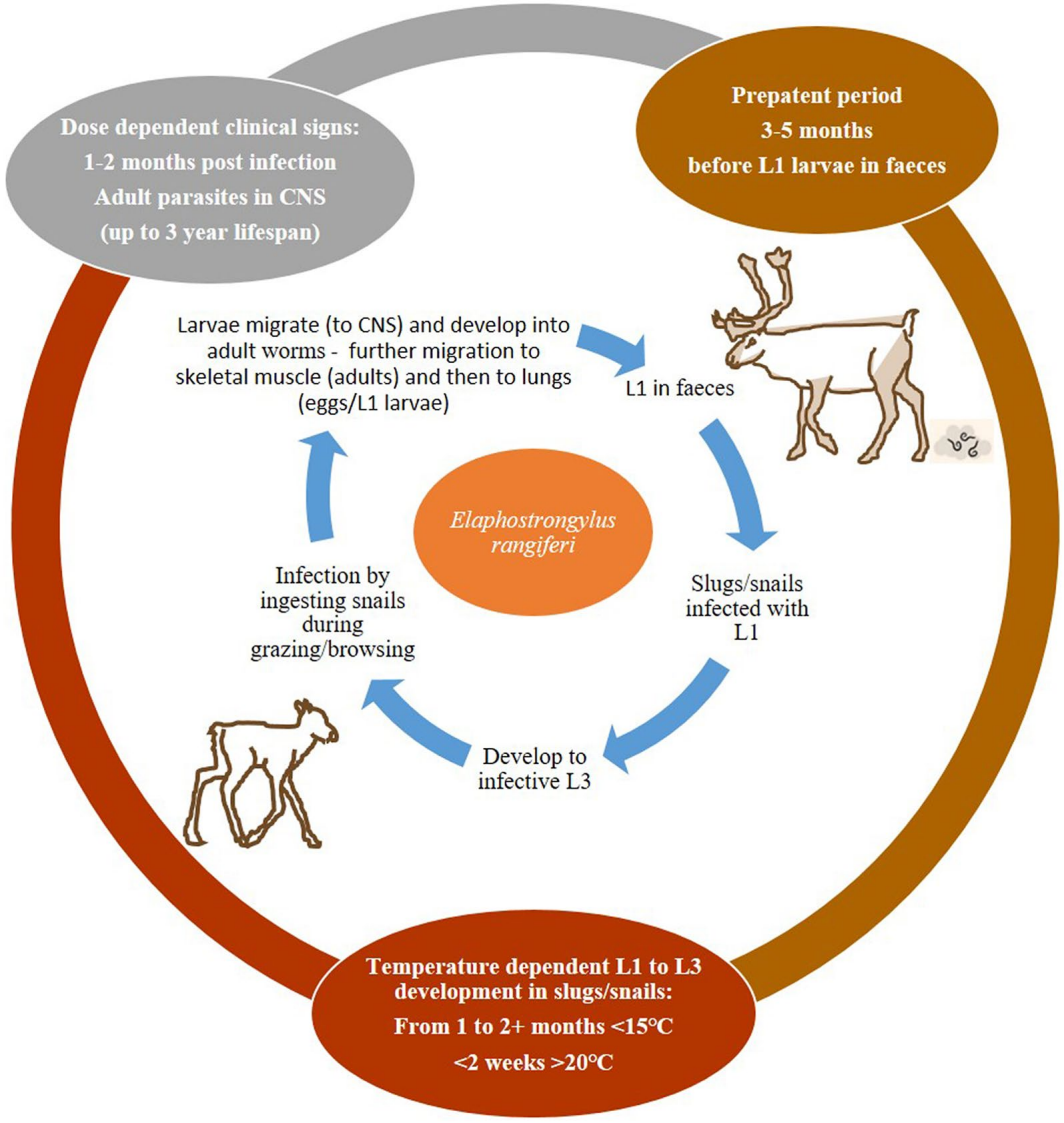
Lifecycle of *Elaphostrongylus rangiferi,* brainworm, with reindeers as the final host and with slugs and snails as the intermediate host

Brainworm has an indirect lifecycle with slug and snail intermediate hosts (Fig. 1). Infection occurs when reindeer consume intermediate hosts containing infective L3 (larvae at third developmental moult stage). After the initial infection, the L3 larvae migrate from the gastrointestinal tract via the vascular system throughout the body. Only larvae which reach the CNS develop further, whilst larva in other tissues die. Once in the CNS the larvae mature from L3 stage to adults from 48 to 90 days post infection (dpi). The parasite is found in the epidural (spinal cord) and subdural (brain) spaces of the CNS [13–15]. Post 90 and until 182 dpi an increasingly higher proportion of adults are found on the skeletal muscles and significantly fewer in the CNS. A small number of adults can continue to be detected in the spinal cord until 196 dpi [13, 15]. The mature nematodes in the skeletal muscle produce eggs that are transported, via the circulatory system, to the lungs. The eggs lodge in the lungs where L1 larvae hatch and then enter the alveoli [14, 16]. These L1 larvae are coughed up, swallowed and passed in the faeces. These findings concur with earlier conclusions that the parasite requires 3–4 months to reach maturity [10] and a prepatent period of approximately 4 months has also been seen in experimental infections [16].

The L1 larvae are very resilient and can survive a year of freezing (at − 80 °C) in water (Table 1) as well as more than 13 months in faeces on pasture, under natural climatic conditions [3, 17, 18]. L1 larvae can infect a number of gastropod hosts and develop to infective L3 larvae. The rate of infection in snails is naturally L1 density dependent in the environment and also varies depending on the age of the L1 larvae [19]. The speed of development from L1 to L3 stages and intensity of infection also varies considerably between gastropod species.

**Table 1:** *Elaphostrongylus rangiferi* L1 larvae survival times in water [17]

Temperature	− 80 °C	− 20 °C	+ 6 °C	+ 12 °C	+18 °C	+ 30 °C	+ 40 °C
Longevity (days)	> 360	> 360	210	150	85	25	8

The following species have proved suitable hosts under experimental conditions: *Discus ruderatus*, *Arion silvaticus*, *Deroceras laeve*, *Euconulus fulvus* and *Trichia hispida*. Other species that successfully developed L3 larvae albeit at a slower rate and lower intensity were *Succinia pfeifferi*, *Deroceras reticulatum*, *Clausilia bidentate* and *Arianta arbustorum*. *Cochlicopa lubrica*, *Arion subfuscus*, *Arion hortensis*, *Vitrina pellucida*, *Nesovitrea* spp. had considerably delayed and extremely poor development and there was no development in *Vertigo lilljeborgi* and *Punctum pygmaeum* [20]. All the species used in that study [20] were collected from the island of Tromsø in northern Norway.

The following four gastropod species, based on the development times and infection intensities seen, would seem to be particularly suited for the development of *Elaphostrongylus* larvae: *Discus ruderatus*, *Arion silvaticus*, *Deroceras laeve* and *Deroceras reticulatum*. The first three species also had the shortest development times.

On reindeer summer pasture in Finnmark (below the treeline) 13 different species of snail and two species of slug were identified with the slug *Arion subfuscus* being the most abundant and widespread whilst *Discus ruderatus* and *Vitrina pellucida* were the most abundant snail species [21]. In Newfoundland the gastropod that dominated collections, and the only one to contain Protostrongylidae larvae, was *Deroceras laeve* at a low prevalence (< 1%; N = 1290) [22]. *Deroceras laeve* slugs have been shown to be active on vegetation until the mean overnight temperature falls below 1 °C [23]. Motile larvae have been detected for up to 6 months in gastropods [3] and larvae remained viable even after the death of the intermediate host. Interestingly, even though gastropods could be infected with L1 larvae in the winter further larval development was delayed by several months, even at room temperatures [3].

### Elaphostrongylosis clinical presentation

The severity of clinical signs depends on the infection dose and the migration paths taken by the parasite in the CNS [15, 16]. Infected reindeer will often mask their clinical signs and only under exercise or during periods of thermal stress (like extreme cold between − 35 and − 45 °C) do the signs become apparent [24, 25].

**Fig. 2:**
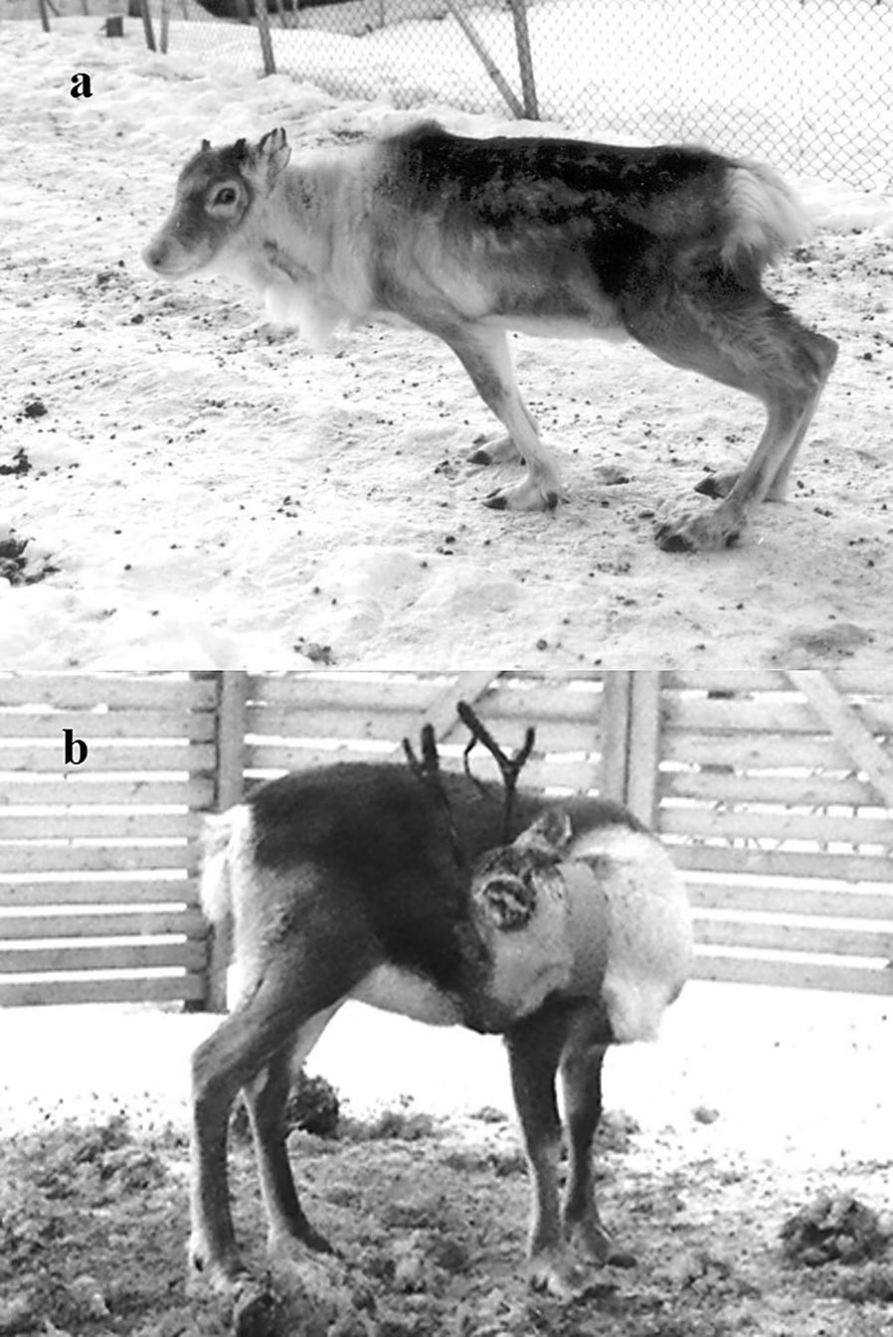
**a** Reindeer showing neurological signs suggestive of clinical elaphostrongylosis with the characteristic kyphotic stance (courtesy of Kjell Handeland); **b** reindeer showing circling behaviour with *Elaphostrongylus* infection (courtesy of Morten Tryland)

The classical clinical signs include general weakness, poor coordination with hindlimb paresis, sometime paralysis, ataxia, knuckling of the carpus/tarsus, stumbling and falling, as well as circling [10, 11, 14, 25]. The stance of the animal is often abnormal with an arched back and sunken down pelvic region (kyphosis), and impaired tail lifting has been reported [16, 25, 26] (Fig. 2). Visual impairment and somnolence are sometimes seen, whilst poor/reduced growth has been recorded in calves. Head tilt was reported in one animal and a live nematode detected on the mucus membrane of the left middle ear of this animal [11]. Clinical signs can take up to 5 months to resolve completely and thus infected animals will have long lasting neurological (and motor) impairment throughout winter, which probably impacts survival chances [16].

Verminous pneumonia is detected in the majority of clinical cases histopathologically at post-mortem. However no clinical signs relating to the respiratory tract have been reported in natural infections [11, 24]. Coughing was reported from one reindeer calf experimentally infected with moose brainworm, *Elaphostrongylus alces,* from 50 dpi [27]. The majority of infections are thought to be asymptomatic, or under detected, given that the prevalence of *E. rangiferi* L1 larvae in adult reindeer can be as high as 60–100% [11, 17, 28, 29].

### Pathogenesis

In experimental studies of *E. rangiferi* infection in reindeer, infective larvae penetrated abomasal venules and spread through the venous system to the right side of the heart. Larvae then reached the lungs via the pulmonary artery. Larvae penetrated the pulmonary venules and were transported to the left side of the heart and then spread throughout the body [15]. This larval migration causes focal necrosis, thrombosis and inflammation in different tissues. Inflammation in the lungs and liver healed quickly while infarcts in renal tissue caused a more persistent interstitial nephritis. Larvae can get trapped in the end arteries of tissue other than CNS. These larvae generally die, however some do survive and continue to migrate to the CNS through the spinal nerves [15].

The development to adults takes place in the parts of CNS which are connected/continuous with the cerebrospinal fluid system. During development, the nematode causes focal degeneration and inflammation of CNS-structures and spinal nerve roots. Mature nematodes leave the CNS from 90 to 196 dpi. The nematodes move from the subarachnoid to the subdural space and then further, via the spinal nerves, to the skeletal muscles [13, 15].

### Neuropathology

In caribou, predominately female nematodes were found in the CNS and, in a few cases, these penetrated up to 1 cm into the brain tissue. The majority, however, were free in the subdural space or loosely attached to the dura or pia-arachnoid [22]. In experimentally infected semi-domesticated reindeer gross pathology showed oedema, discoloration, petechial and ecchymotic haemorrhages as well as the presence of mature nematodes in subdural space of spinal cord and brain and dural sheets of spinal nerve roots [15].

**Fig. 3:**
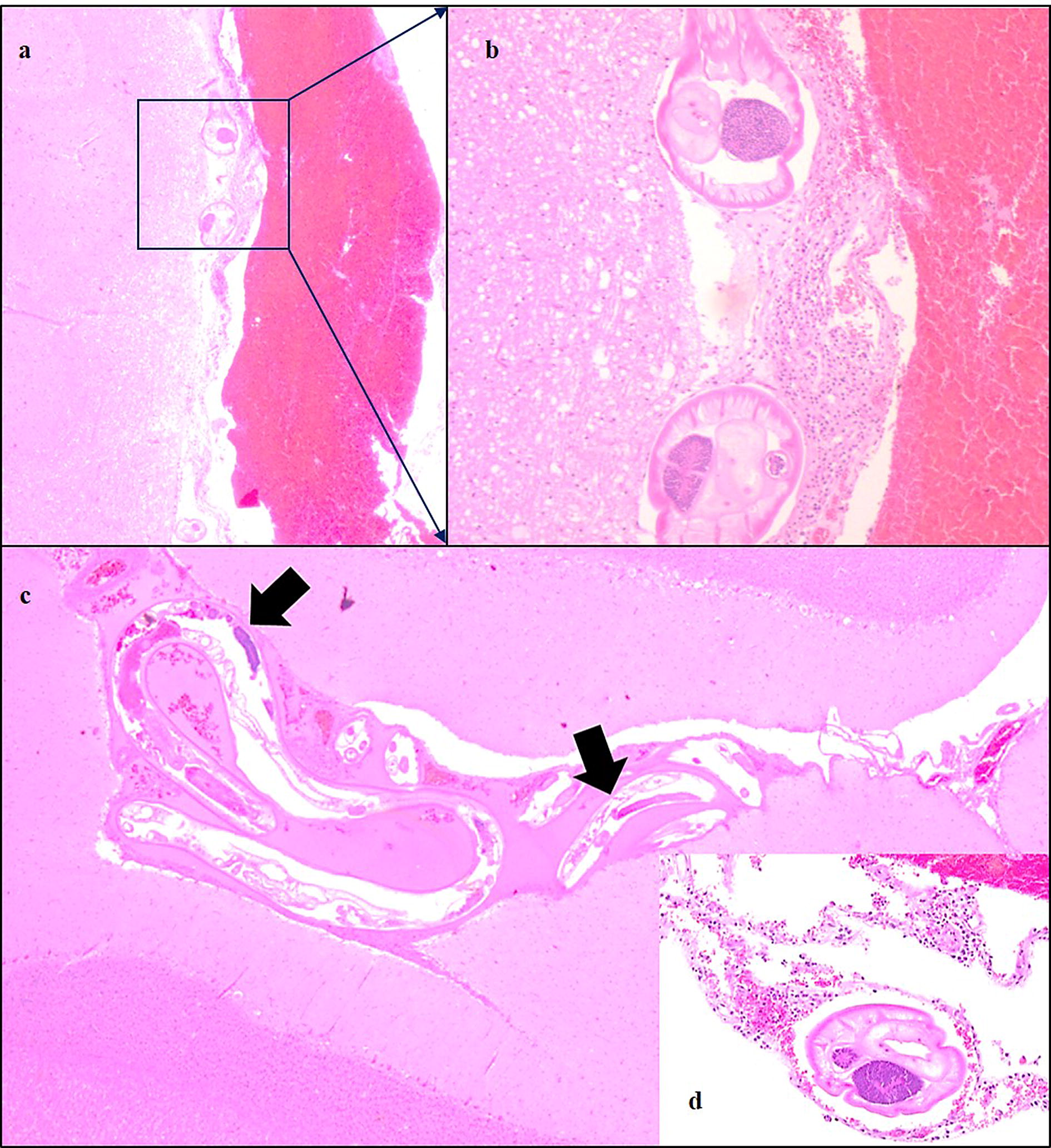
Photomicrographs showing histopathological changes in the brain of a reindeer infected with *Elaphostrongylus rangiferi***a** Multiple nematodes and associated inflammation in the meninges covering the cerebellum. The haemorrhage was caused by the euthanasia (Obj. 2.5×). (**b**) Detail of **a** showing the cellular infiltrate around the nematodes (Obj. 20×). **c** Longitudinal sections of nematodes (*black arrows*) (Obj. 10×). **d** Transverse section of a nematode and mononuclear cell response in the meninges of the cerebellum (Obj. 20×). **a**–**d** haematoxylin and eosin

Histopathological findings were more often detected in the spinal cord than in the brain. Nematodes were found in the meninges whilst inflammation was seen in the meninges, the CNS parenchyma, central canal and brain ventricles (Fig. 3). In the meninges, the inflammation consisted of eosinophil, neutrophil and mononuclear cellular infiltration. Small granulomas encircling nematode debris were sometimes found, mainly in the subarachnoid space. In the parenchyma, perivascular cuffs with eosinophilic or mononuclear cells were found in both the brain and the spinal cord. Malacic foci with cell debris, eosinophils and macrophages and degenerated axons caused by nematodes migrating in the spinal cord parenchyma, were seen. Nematodes and inflammatory cells were also found in central canal of spinal cord and in the brain ventricles.

Nematodes were found in the perineureum and in some spinal nerve roots. Histopathological findings were focal degeneration with axon and myelin sheath necrosis and an inflammatory reaction similar to that seen in the CNS [15].

### Muscle pathology

**Fig. 4:**
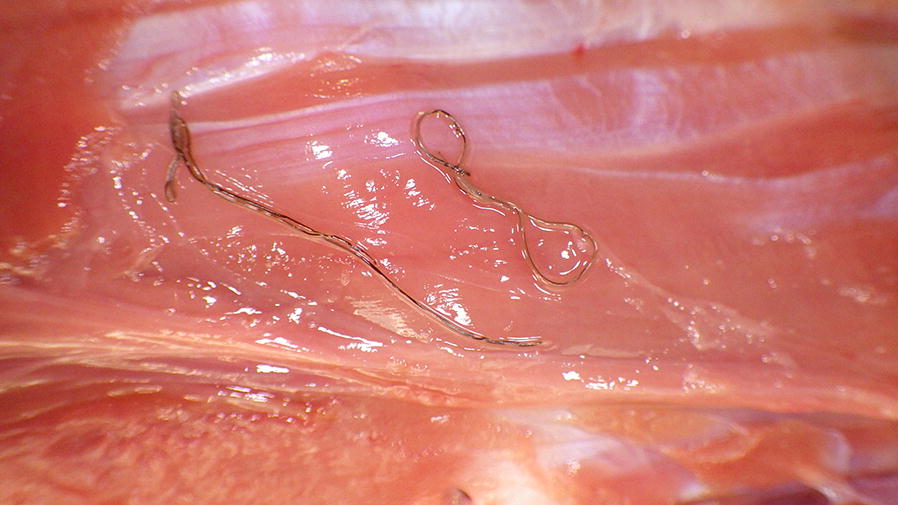
Adult *Elaphostrongylus* nematodes in muscle fascia (Courtesy of Kjell Handeland). The thin dark threadlike parasites can be seen coiled in the centre of the image

Adult *Elaphostrongylus* in the skeletal muscles are generally found close to the nerve root, with the latissimus dorsi, obliquus externus, longissimus dorsi muscles overrepresented [13]. Inflammation and intermuscular oedema may be the first noticeable sign before closer examination reveals the presence of the adult nematode [9, 15]. Green discolouration of connective tissue and muscle fascia has been reported at meat inspection and closer inspection of these areas will often reveal a small black coiled hair-thin parasite, about 3–7 cm long, in the intermuscular layers and subcutaneous connective tissue (Fig. 4) [9]. In years with particularly high infection pressure, partial or complete condemnation of the carcass may be required [9].

### Lung pathology

**Fig. 5:**
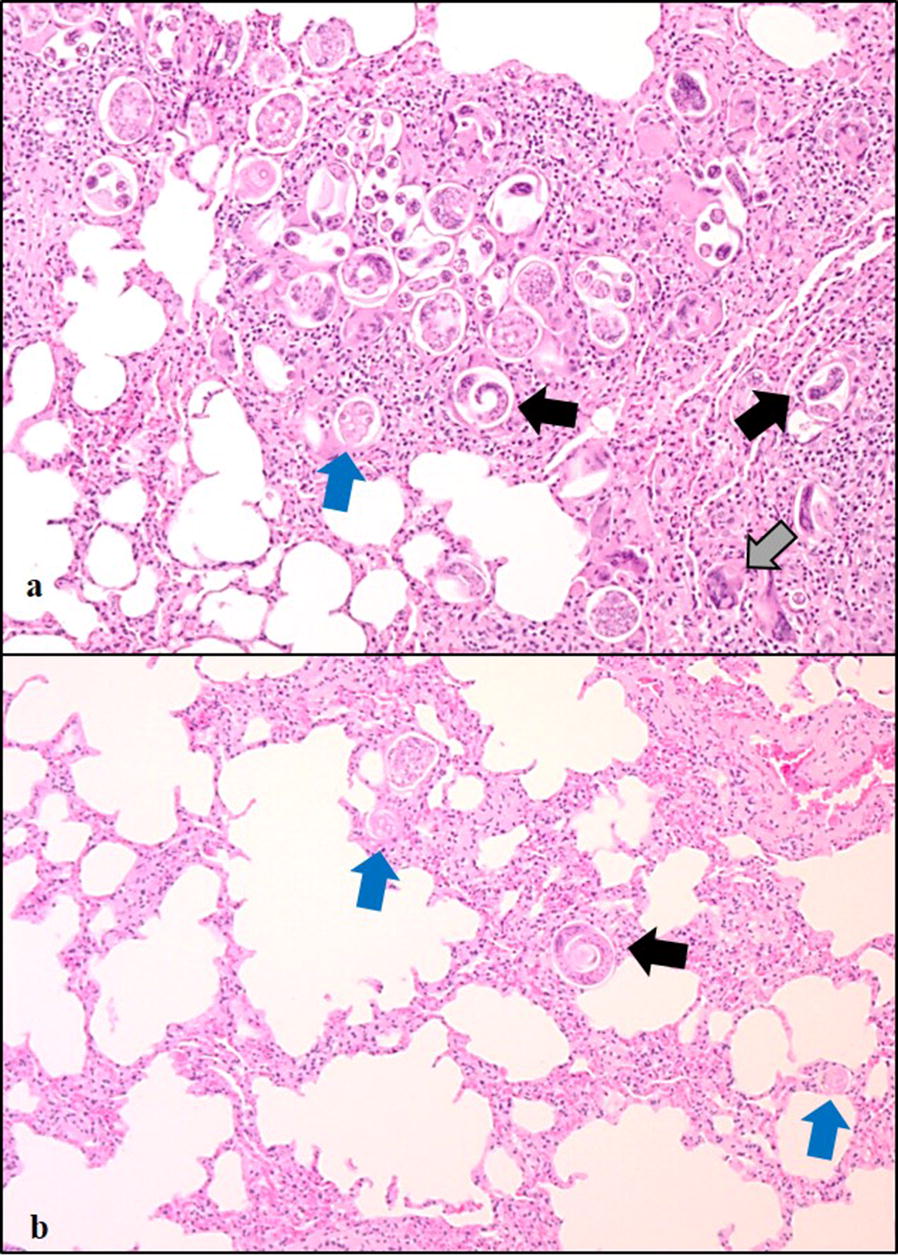
Photomicrographs showing histopathological changes in the lungs of a reindeer infected with *Elaphostrongylus rangiferi*. **a** Heavily infected area with multiple transverse as well as longitudinal larvae (black arrows) and eggs (blue arrow). Mononuclear cells and giant cells (grey arrow) surround the parasites. **b** An area less infected. A coiled larva (black arrow) as well as several eggs (blue arrows) are seen. Mononuclear cells are present in the interstitium. **a**, **b** Obj. 10×, haematoxylin and eosin

Eggs from mature nematodes are transported via veins to the lung where they cause a chronic interstitial pneumonia (Fig. 5). In most cases there seems to be a multifocal mild inflammatory response with few leukocytes, mainly mononuclear cells, surrounding the eggs and/or larvae. Small granulomas may also be found in the alveolar septa. However, in more heavy infections, with large numbers of eggs and larvae, a diffuse and more severe inflammation is reported [1, 15].

### Diagnosis

Clinical signs generally develop before larvae can be detected in the faeces [13, 16]. The primary diagnosis therefore has to be based on classic clinical neurological signs combined with confirmatory gross and histopathological findings.

**Fig. 6:**
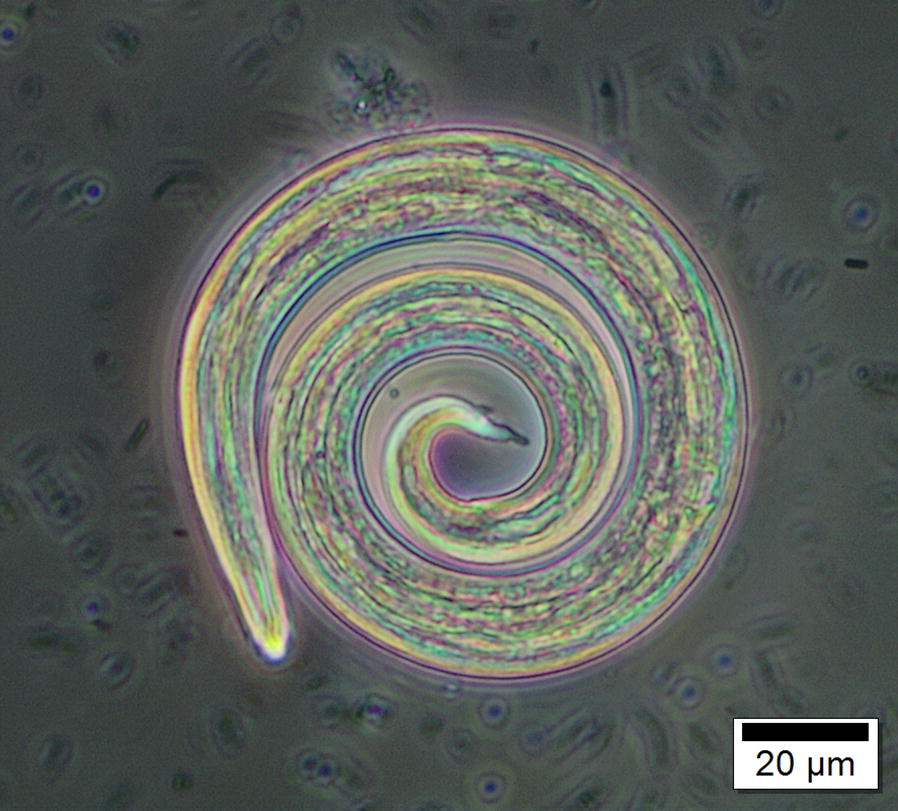
*Elaphostrongylus rangiferi* larva at L1 development stage. The larva has been fixed in ethanol prior to photography

Baermann’s analysis, to isolate faecal larvae, can be used to investigate brainworm larval prevalence and abundance in a herd [30]. The L1 *Elaphostrongylus* larvae have a distinctive s-shaped tail with a dorsal spine common to many protostrongylids (Fig. 6). Measuring larval length and looking at the morphology of the tail and dorsal spike can help discriminate between different protostrongylid species (*Elaphostrongylus* spp., *Varestrongylus* spp., *Muellerius capillaris*) (Table 2) [31, 32].

**Table 2:** Sizes of different Protostrongylid L1 larvae with a dorsal spine in Fennoscandian wild ruminants that have been found in natural infections in these hosts [31, 32]

L1 DSL larvae	Body length (µm)	Body width (µm)	Hosts
*Muellerius capillaris*	250–320	12–15	Sheep, goats, muskoxen, caribou
*Elaphostrongylus rangiferi*	381–490	17–24	Reindeer, caribou
*E. cervi*	392–445	17–22	Red deer
*E. alces*	377–445	17–21	Moose
*Varestrongylus alces*	221–374	12–30	Moose
*V. sagittatus*	260–305	8–16	Red deer
*V. capreoli*	255–341	10–17	Roe deer

The adult parasites can be found as thin threadlike nematodes in the subdural and epidural surfaces of the CNS as well as in connective tissue between the muscles. *Elaphostrongylus rangiferi* can be differentiated from the other *Elaphostrongylus* species found in wild cervids in Fennoscandia (*E. cervi* and *E. alces*) based on morphological differences in the length and morphology of the bursal rays, spicules, genital cone and gubernaculum in males as well as female body length, width and shape of the tail. Adult females are from 3.3 to 7 cm long, and approximately 100 µm wide, with a bluntly rounded tail [9, 33, 34]. Adult males are between 2.6 and 3.5 cm long and approximately 100 µm wide with a bluntly rounded anterior. The bursa has a bell-shaped appearance, the short lobes are not separated and the dorsal ray is divided in two, with a short joined base, and two branches each [27, 33]. Males have two sub-equal spicules, a rod-like gubernaculum, and the genital cone has a ventral cone-shape with two dorsal papillae [33, 34].

### Epidemiological considerations

There is large variation in annual prevalence in calves and yearlings and parasite L1 excretion prevalence increases with increasing age [17, 28]. Within herd prevalence can vary considerably during the year from 40% in summer to 100% in winter [29]. The intensity of larval output also varied considerably during the year with the highest larval faecal densities recorded in spring (April) and the lowest in summer (July) for one herd in Karasjok, Norway [29].

**Fig. 7:**
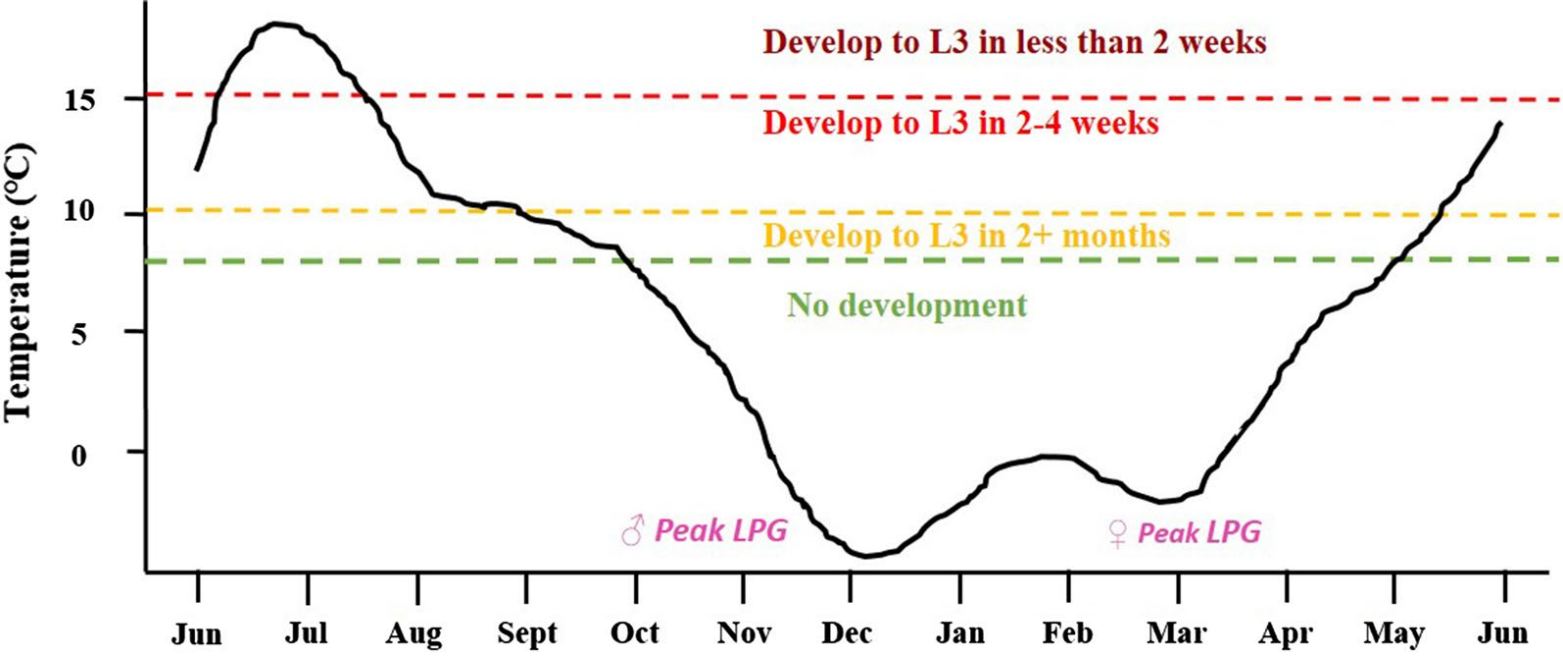
Time taken for *Elaphostrongylus rangiferi* L1 larvae to develop to L3 at different temperatures. Temperature data for Trøndelag, Norway was used to show fluctuations in mean monthly environmental temperature (for 2018–2019; data from http://www.yr.no) and the developmental threshold times indicated [50] (reproduced with permission of Dr. Hannah Vineer, University of Liverpool). Also shown are the peak periods for brainworm L1 larval output by male and female reindeer [29]

Seasonal differences in L1 larval output have been recorded with different peak excretion times depending on definitive host gender [29]. There is minimal larval output during mid-summer (July–August) for both sexes, whilst female reindeer have maximum output in late winter and spring (February–April) and male reindeer show maximum output in autumn/early winter (October–December) (Fig. 7). This gender related seasonality most likely reflects immune related responses to the rut in males (in autumn) and to calving in females (in late winter) rather than parasite regulated seasonality [35]. The lifespan of an adult brainworm nematode was estimated to be 3 years [29].

Study of the free-living larval L1 larval stage has shown that it can survive repeated desiccation and rehydration episodes for up to 4 months, although the larvae were non-motile after the first desiccation event [3]. These immotile larvae however became motile again after rehydration. Mitskevich [3] observed that all free-living L1 larvae, regardless of when obtained from faeces, were motile until December. The larvae then entered non-motile states until April—even when kept at room temperature. The L1 larvae in faeces were able to persist, albeit with decreasing intensity, for more than 2 years under natural conditions (freezing, thawing, sunlight and precipitation). These observations have not been reported elsewhere and would need further verification.

Infected reindeer calves were heavier than uninfected [36]. This was posited to be a result of dominant individuals being larger and thus having greater access to preferred foods like calcium rich herbs, thereby having a higher chance of ingesting infected gastropods than lower ranking herd members. In years without disease outbreaks, *Elaphostrongylus* infection occurred mostly in the large male calves. A similar trend with size and infection levels was also seen in female calves, with the largest females having a higher prevalence than the smaller females. This gender bias was also seen in caribou in Newfoundland where 2/3 of the animals with clinical elaphostrongylosis in 1981–1985 were males [22].

### Infection and disease in other species

Experimental studies as well as diagnostics of clinical cases have shown that sheep and goats, grazing in the same areas as reindeer, are susceptible to *E. rangiferi* infections. The parasite is able to infect and complete migration to the CNS in both sheep and goat, but fails to mature further and does not reach the skeletal muscle [1, 37, 38].

**Fig. 8:**
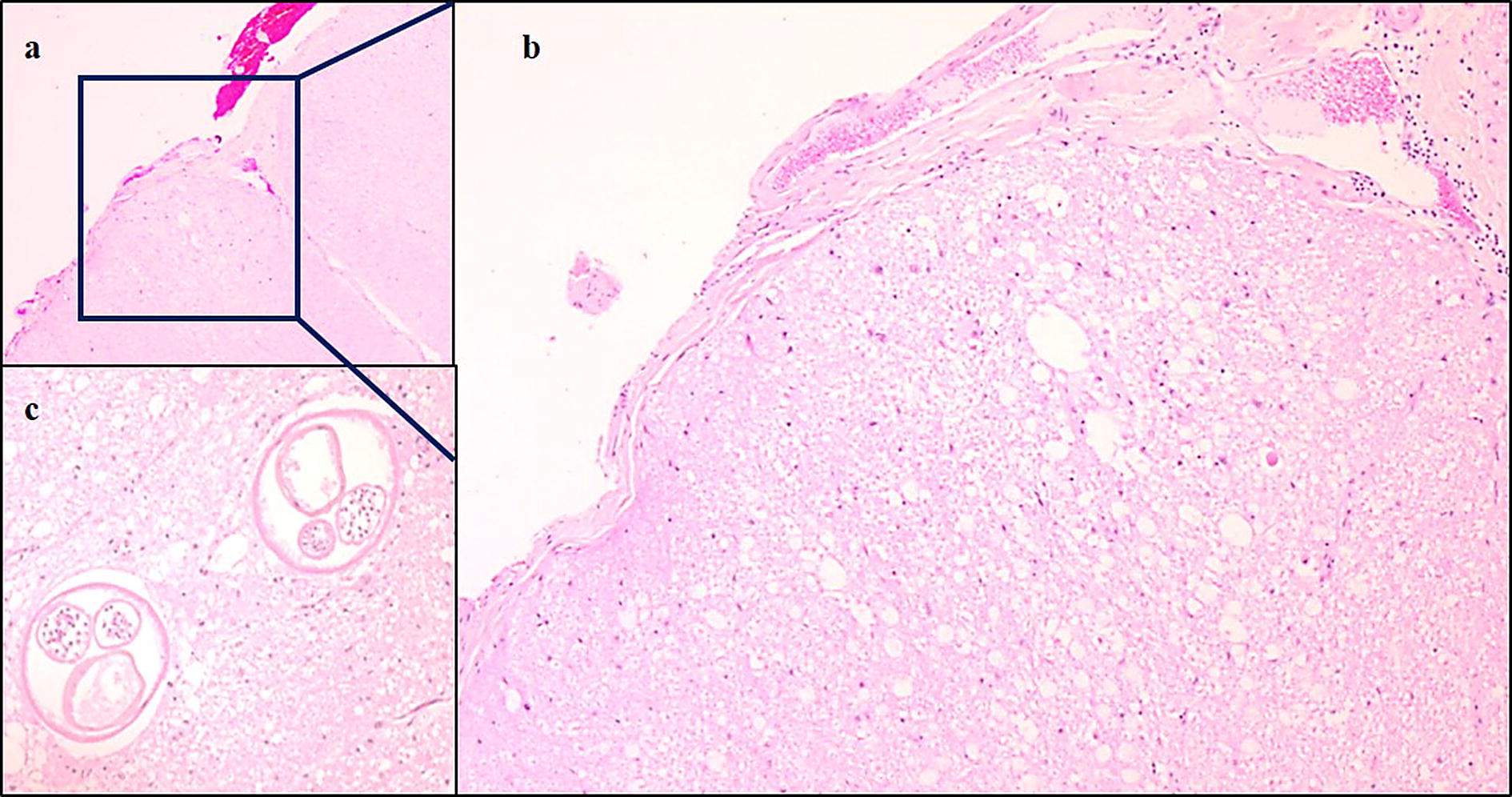
Photomicrographs showing histopathological changes in the spinal cord of a goat infected with *Elaphostrongylus rangiferi*. **a**, **b** foci of malacia in spinal cord white matter due to nematode migration. **c** Transverse sections of adult nematodes in the white matter. **a**: Obj. 2.5×; **b:** Obj. 10×; **c**: Obj. 20×. **a**–**c**: Haematoxylin and eosin

In both sheep and goats, the clinical signs of disease first appear 1–3 months after infection. Typical symptoms are ataxia and hind leg paresis and more rarely brain disturbances. In addition, itching and pruritus causing varying degree of hair loss and skin lacerations are seen before or simultaneously with CNS signs. The histopathological findings in the CNS resemble those found in reindeer, with the additional presence of granulomas surrounding dead nematodes (Fig. 8). Sheep only occasionally develop disease after infection while goats seem to be more susceptible [38–40].

Posterior ataxia was reported in five muskoxen (*Ovibos moschatus*) on the Norwegian-Swedish border in 1988 and the animals were euthanised. Histopathological examination of the CNS from these animals identified leptomeningitis in the caudal lumbar and sacral regions of the spinal cord [41] with nematodes found between the dura mater and neuroparenchyma. The histopathological findings were very similar to those seen in goats and sheep. Fragments of immature nematodes were found in gelatinous foci on the spinal cord. Definitive species identification was not carried out but given overlapping grazing with reindeer and moose it was concluded that *Elaphostrongylus* sp. was the culprit [41]. Experimental studies have shown that although moose (*Alces alces*) can be cross-infected with *E. rangiferi* and reindeer with *E. alces* the reproductive ability of the parasites in the alternate host species was severely reduced [27]. The results of the study also indicated that *E. rangiferi* can be highly pathogenic in other cervid hosts. Two of the three moose infected with *E. rangiferi* became moribund and were euthanised before the lifecycle could be completed (< 95 days). The third died 5 months after the initial infection with 1000 larvae. L1 larvae were detectable in the faeces from 133 dpi. The clinical signs reported in all the infected moose included anorexia, fever, pain and neurological deficits [27].

### Documented outbreaks

We have limited reports of disease incidence and prevalence from reindeer herders and no long-term baseline prevalence or abundance data. In a study from northern USSR between 21 and 60% of reindeer faecal samples contained *Elaphostrongylus* larvae [11]. Bakken [24] mentions disease outbreaks in Sweden and USSR after the parasite was first described in 1958 but no further details are provided.

### Norway

The earliest reports of clinical elaphostrongylosis in scientific literature from Norway are from the 1960’s with the first case in Pasvikdalen in 1960 followed by an epizootic in 1960–1961 in south Varanger (Finnmark, northern Norway) [9]. The following year, 1961–1962, more calves were reported to have succumbed in south Varanger. One herd lost around 500–600 calves (of 900 in total) during the course of the winter. Reports suggest between 60 and 70% of the calves in affected herds succumbed [42]. Elaphostrongylosis was diagnosed based on the clinical signs alone [9]. Those with the most severe clinical signs died first whilst those with a milder course struggled during the winter before succumbing [9]. Individual cases continued to be reported from the Varanger district [9] in 1965 and 1967, some of which were confirmed as elaphostrongylosis by post-mortem (gross and histological diagnosis) but no large scale outbreaks were registered. Adult *E. rangiferi* was recorded in muscles at meat inspection during the autumn slaughtering period in 1964, 1965 and 1968. The parasites seen on the muscles were confirmed morphologically as *E. rangiferi*. *Elaphostrongylus* was detected in 1.1% of the 4736 carcasses inspected in 1968—four carcasses had to be fully or partially condemned. Grøholt [9] suggested that the inherent difficulties of meat inspection in the field, like frozen carcasses, poor lighting and other suboptimal conditions, probably resulted in a significant underestimation of infection prevalence. Grøholt [9] suggested that herds grazing in the mountain areas around the Alta-fjord in summer (especially Kåfjord, Talvik and Bognelv) had the most muscle parasites at slaughter in the autumn whilst those grazing at higher altitudes away from the coast seldom had the parasite (Bæskades, Nassa, Nabar, Sennaland and the areas towards Stabbursdalen and Transfarelv (Seinos)). A more moderate prevalence was seen in mountain areas around eastern-Talvik, Øksfjord peninsula (Jøkelfjord and Øksfjord) and the Porsanger peninsula.

The next major outbreaks were reported in Finnmark in the early 1970s [10, 24–26, 42, 43]. The climatic conditions were highlighted as being the trigger given a mean summer temperature almost 2 °C warmer in years with outbreaks (13.4 °C) than those without (11.5 °C) [10, 42]. Clinical elaphostrongylosis was common in eastern and western Finnmark during the five winters of 1970/71 to 1974/75 [42]. The total loss of reindeer in winter 1970/71 was estimated to be in the thousands. This outbreak officially began in January 1971 with reports of more than 100 dead animals in Gavnejavrre (80–100 km southeast of Kautokeino) and subsequently a large number of reports of sick and dying animals from multiple herds across the district [24]. The clinical signs reported were head shaking, ataxia, limb paresis and paralysis especially after exertion. Elaphostrongylosis was confirmed at post-mortem (gross and histopathological changes) in 11 of 19 carcasses [24, 25]. Meat inspection results from a subsample of animals later that year found a tenfold higher prevalence (11% of 79 carcasses) of muscle nematodes than studies 5 years earlier [9, 24]. In the next recorded outbreak, the first cases of clinical elaphostrongylosis were reported in August 1972, with affected calves on summer coastal pastures in Finnmark [10, 26]. The first part of the summer in 1972 had been particularly hot. The diagnosis was confirmed histopathologically on a small subsample. Herders reported that the entire generation of yearlings died that following winter from general weakness [10] likely as a result of chronic disease and reduced foraging ability at a time of year (late autumn and winter) when food resources are harder to obtain [42].

No further outbreaks were reported in the literature prior to 2018. The Norwegian Veterinary Institute received a report regarding sick and dying calves from a herd in Trøndelag (central Norway) in August 2018 [44]. Other age groups also sickened as autumn progressed. Despite the provision of supplemental feeding and close monitoring of the herd about 70 animals died. According to the herder many of the calves that had survived the initial outbreak perished during the subsequent winter. The following year the herder moved the flock to the high mountain pastures as early as possible and preliminary reports suggest that this strategy may have been successful compared with other herds from this reindeer herding district that chose to keep their reindeer on lower pastures (unpublished data).

**Fig. 9:**
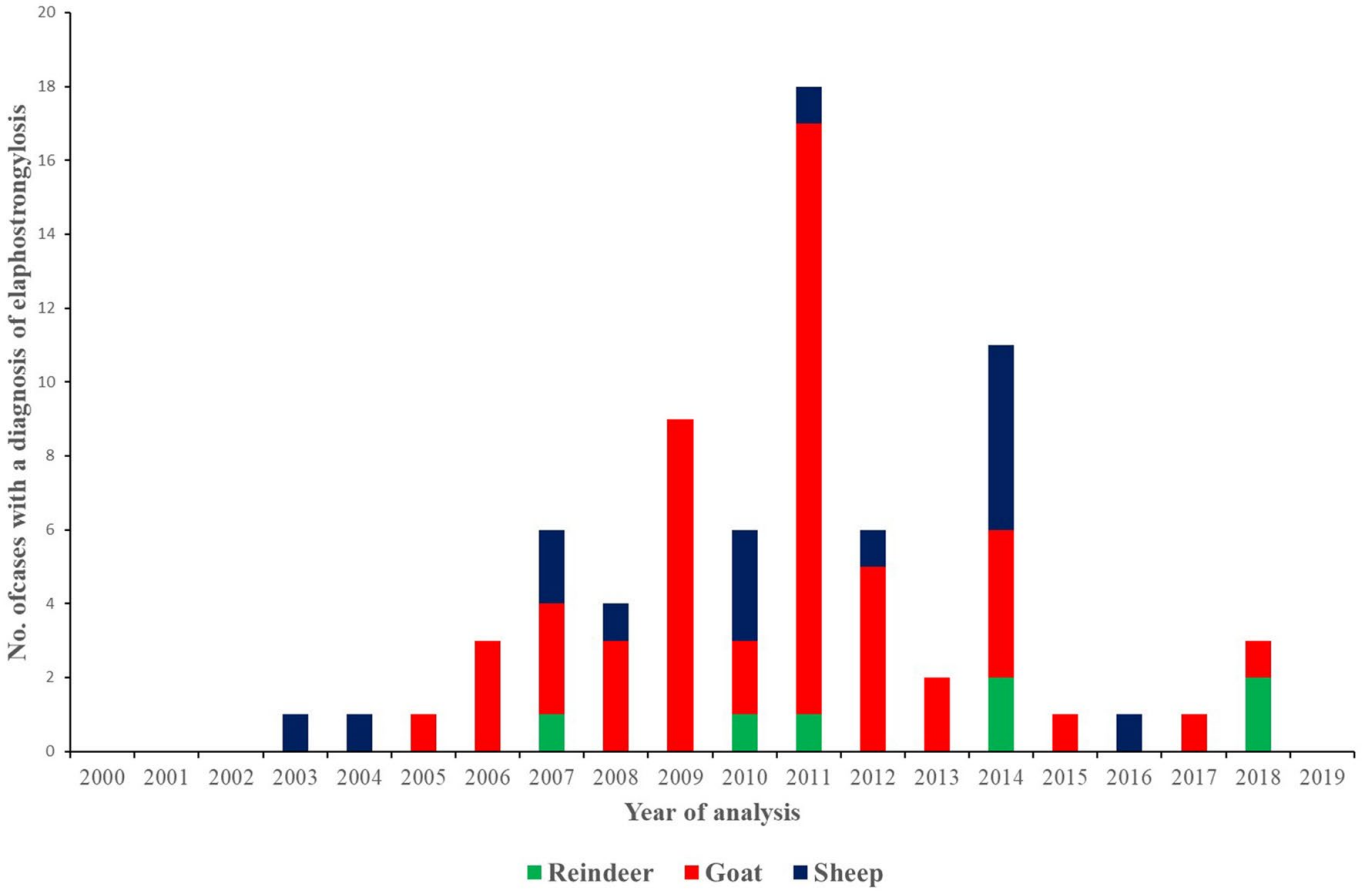
The number of elaphostrongylosis cases in the period 2000–2019 diagnosed at the Norwegian Veterinary Institute by species (reindeer, goat, sheep). The diagnosis is based on the detection of histopathological changes and nematodes in the central nervous system combined with the presence of clinical signs. However the species of *Elaphostrongylus* involved was not further identified. We cannot rule out that some of the small ruminant cases in particular could potentially be caused by other brainworm species like *E. cervi* (from red deer) or *E. alces* (from moose) especially in areas where grazing does not overlap with reindeer

A survey of the diagnoses recorded at the Norwegian Veterinary Institute during the period 2000–2019 (until end of November) reveal that this disease is still very much prevalent. *Elaphostrongylus* infections and/or pathological changes suggestive of elaphostrongylosis have been confirmed in 199 animals (131 reindeer, 51 goats and 17 sheep) during the last two decades. Seventy-four cases of confirmed clinical elaphostrongylosis (Fig. 9) have been recorded in goats (n = 51), sheep (n = 16) and reindeer (n = 7). However, deductions regarding annual variations in disease/parasite diagnosis cannot be made based on this dataset. The animals investigated have been submitted for a wide range of reasons; routine diagnostics, research projects, targeted disease investigation. In many cases only a few animals from a herd are submitted for further post-mortem and laboratory investigation. For example, just three calves had post-mortem investigations carried out in the 2018 outbreak in Trøndelag. The total number of small ruminants submitted for post-mortem to the Norwegian Veterinary Institute in northern Norway significantly reduced in 2012 as the cost for this service increased substantially and, with farmers and veterinarians more familiar with this disease, post-mortem was no longer requested.

### Sweden

Very little information is available about the situation in Sweden although both the parasite and disease have been seen in Swedish reindeer [45]. High calf mortality due to unknown disease was reported in 1969–1973 [46] but neither aetiological agent nor clinical signs were reported. A survey of reindeer herders in 2006 showed that nearly all herders (98%; n = 40) were aware of the disease and that treatment with ivermectin once a year for parasites, in late autumn/winter, was assumed to be effective against brainworm [47].

### Finland

No major outbreak of reindeer elaphostrongylosis has been reported from Finland, but the parasite has been recorded in eastern Lapland [48]. In the Kaamanen experimental reindeer herd in northern Lapland, *Elaphostrongylus* larvae are sporadically seen in reindeer faeces [49].

In the necropsy material of the Finnish Food Authority (previously Finnish Food Safety Authority Evira), between 2010 and 2017, nine reindeer were either found infected by *Elaphostrongylus* or having an anamnestic history and lesions suggestive of elaphostrongylosis. They were sporadic cases and mostly solitary animals from their respective herds.

Only one of the four gastropod species, *D. ruderatus*, suggested as good intermediate hosts (*D. ruderatus*, *A. silvaticus*, *D. laeve* and *D. reticulatum*), has commonly been found in the Finnish reindeer husbandry area (https://laji.fi/en).

### *Elaphostrongylus* and climate

Further work has been carried out looking at where in the reindeer grazing areas the most gastropods can be found, how infection with L1, L2 and L3 larvae influences snail overwintering survival and temperature dependent development times in two snail species *A. arbustorum* and *E. fulvus* [17, 50–52]. A developmental zero temperature of between 8 and 10 °C was seen [50, 51]. The authors concluded that with average summer temperatures in northern Fennoscandia, the majority of L1 larvae shed on the pasture during the summer grazing season would not complete development to L3 in gastropods prior to the onset of winter and snow fall. Therefore, *Elaphostrongylus* relies on overwintering in adult gastropods and has a 2-year lifecycle. The extreme freeze tolerance of the L1 larvae suggests that these could also successfully overwinter as free-living stages and snails infected with L1 larvae had significantly better winter survival rates than snails infected with L2 and L3 larvae [52]. Whichever strategy predominates, climate will influence larval survival and development as well as intermediate host survival.

Warmer temperatures result in faster penetration of gastropods by L1 larvae, more rapid larval development times (Fig. 7), from 2 to 4 months to less than 2 weeks, resulting in increased L3 infection intensity in snails [50, 51]. The earliest recorded clinical signs have been reported in August in Finnmark (1972) and Trøndelag (2018) [10, 44]. This suggests that large numbers of infective L3 in gastropods were consumed early in the season, at least 4–6 weeks previously (from June) either as overwintering infectious snails or after rapid development to L3 in the spring.

Precipitation effects on infection pressure are less clear [42, 53]. Halvorsen [53] found no significant effects between summer precipitation and reindeer density and brainworm abundance in faecal samples whilst Handeland et al. [42] reported an association between increased summer rainfall and disease outbreaks. Both studies saw a significant correlation between increasing summer temperature and increasing parasite abundance/disease outbreaks. Given the different age groups investigated, Halvorsen [53] also explored whether a lag from infections in earlier summers (up to 2 years previously) could influence abundance. His data showed no such lag.

Infection pressure is also influenced by where the reindeer graze during different seasons. Snail densities were highest below the timberline in one reindeer grazing area [17]. Reindeer only grazed a relatively short period below the tree line during summer since insect harassment drove the animals to higher pastures as the snow melted. However, some gastropods were found in calcium rich bogs above the timberline in this study area (Porsanger, Finnmark, Norway) whilst gastropods were scarce on winter grazing predominated by lichens [17]. Infections would seem to occur during summer grazing, given limited infection risk along migration routes due to the timing of migration and seasonal weather conditions (snow cover), combined with the presence of faecal larvae in samples from calves from early autumn. The greatest risks of infection therefore come from grazing calcium rich bogs above the treeline as well as any grazing below the treeline. This is supported by findings in wild reindeer in Norway. Animals with summer grazing at higher altitudes had lower brainworm prevalence [30].

Earlier spring and higher spring and summer temperatures, because of climate change, may well impact on intermediate host distribution and availability to reindeer as well as on the prevalence and abundance of infective larvae in intermediate gastropod hosts. Changes in precipitation may impact infection levels both positively (less snow coverage resulting in earlier availability of gastropod laced grazing) and negatively (desiccation of free living L1 larval stages) [53]. However modelling of infection risk also needs to take into account host sheltering factors like gastropod behavioural responses to thermal extremes [54]. This could buffer the negative effects of extreme heat on the intermediate parasite stages. The gastropod *D. laeve* can preferentially select microhabitats with temperatures below 21 °C and larvae infecting the slug are thus maintained within the optimal thermal fitness range despite tundra surface temperatures being considerably higher [55]. Changing landscape use, timing of flock migration between the different seasonal grazing areas as well as other herding practices, like the use of anthelmintics and fencing could also influence infection risk [43, 56].

### Treatment

Ivermectin and doramectin are currently the only anthelmintics licensed for use in reindeer in Fennoscandia. A number of studies have investigated the efficacy of ivermectin against *E. rangiferi* with varying success. Folstad et al. [57] report that, “as expected”, ivermectin had no effect against *E. rangiferi* given that L1 larvae continued to be detected in the both the treated and control groups during their study. Macrocyclic lactones, like ivermectin, penetrate the blood–brain barrier poorly [58]. However ivermectin (at 200 µg/kg, subcutaneous injection) has been shown to have a moderate effect on brainworm infection levels by reducing larval burden in the lungs by 94% compared to untreated controls [56]. Larval shedding continued throughout the study period despite the reduction, but not elimination, of larval burden in the lungs. Mebendazole and febendazole treatment (6 mg/kg bodyweight, orally, once daily for 10 days) were successful at eliminating faecal larval shedding by 50 days post treatment [56]. However one of the febendazole study animals had pathological evidence of CNS infection 50 days post treatment. These studies were carried out on naturally infected reindeer calves (n = 6 in each treatment group) and the findings require further corroboration.

### Measuring the impact of infection on herds

Climatic limitations on the development of infectious stages and access to ample high quality grazing (maximising potential for animals to develop optimal immune response to infections) contribute to limiting infection pressure and thus epizootics are a rarity rather than the norm. The consensus is that low infection doses result in little to no clinical signs whereas high infection doses can result in a fatal course. From a broader one-health approach, the overall resilience of the herd can be impacted at sub-clinical disease levels.

We currently know too little about baseline infection and disease occurrence to draw any kind of conclusion about the current impact of infection on herd health and resilience. The limited knowledge we have from individual herds, in different regions with differing management practices, is insufficient to provide guidelines for the industry as a whole. We are reliant on the herders to self-report disease outbreaks. The most common reported cause for losses is predators and not disease [8]. Obtaining reliable baseline data on disease incidence, morbidity and mortality is therefore challenging.

### Knowledge gaps and future needs

We currently do not know how much climate change will affect brainworm abundance and prevalence. We anticipate that warmer temperatures will increase larval gastropod infection rates and shorten larval development times. Climate factors that can decrease infection risk would be factors that are detrimental to the survival of the gastropod hosts and the free-living larval stages, such as desiccation and repeated freeze–thaw events, whereas a warmer and wetter climate would benefit both the intermediate hosts and larval development. Anthropogenic impacts on reindeer herding may also result in changing infection risk by reducing grazing flexibility. We lack knowledge about spatial and temporal differences in infection risk which would allow us to advise herders how best to mitigate infection in years with high infection risk. As previously highlighted, we know too little about the impact that disease and infection has not only at an individual level but also at the herd level. Are, for example, reindeer with moderate or high levels of respiratory tract infection (from brainworm) less resilient to predation?

We need more information about what is happening both in reindeer and in the gastropods. How is climate change affecting parasite transmission, reproduction rates and survival? Given the number of recent years that have broken temperature records, we have to ask why are we not seeing more outbreaks? One approach to answer this could be the modelling of climate data in the reindeer regions to if temperatures were favourable for outbreaks in these districts [42] and compare this with information about herding practices and anthelmintic usage, to look for trends. There seems to be considerable geographic variation in disease reporting. Whether this reflects actual differences in infection pressure, presence and absence of suitable gastropods, or different herding practices in the three Fennoscandian countries with reindeer herding or differences in disease reporting mechanisms is hard to say. Further investigation is therefore needed to explain why Norway still sees outbreaks whereas so few are reported from Finland and Sweden.

We also lack knowledge about appropriate mitigation strategies. Different grazing strategies to avoid areas with highest infection risk are one means of reducing morbidity. Another would be to adjust culling strategies in years with high infection risk, as is sometimes implemented in Finland to mitigate for *Setaria tundra* outbreaks following two consecutive years of warm summers. The development of predictive climate and disease risk models is needed to enable pre-emptive parasite treatments or other mitigation strategies for geographical areas (and seasons) with high infection risk. We also need to develop treatment guidelines regarding how best to prevent and to treat a disease outbreak.

## Conclusions

*Elaphostrongylus rangiferi* is a ubiquitous reindeer parasite that can, under the right climatic conditions, cause considerably morbidity and mortality in reindeer herds. The parasite is particularly adapted to survival during winter conditions and warmer and wetter summers will increase transmission risk. But climate change is not the only risk. The impact of anthropogenic changes on the natural resources available to the reindeer, the knock-on effects to herding practices and subsequent loss of grazing flexibility can also magnify the risk of infection. Reindeer herders need to have good awareness of the spatial and climatic triggers that can increase infection risk to understand how best to avoid high levels of infection and how to mitigate disease outbreaks in years with favourable climatic factors. Further research into current herding practices and documenting traditional knowledge, before it is lost, in addition to exploring the presence and prevalence of infection in intermediate hosts and final hosts is needed to obtain this spatial and climatic awareness. Diagnosis of clinical elaphostrongylosis is a combination of detecting clinical signs suggestive of the disease combined with confirmatory histopathological findings in selected individuals. Reindeer herders and veterinarians treating reindeer with neurological signs must take into consideration that faecal L1 larvae are not generally present when clinical signs first appear, thus complicating the diagnosis. There are currently no treatment options available that are suitable for use with extensive reindeer husbandry practices.

## Data Availability

The datasets used and analysed during the current study are available from the corresponding author on reasonable request.

## Prior publication

Data have not been published previously.
